# Anti-Nogo-A antibody treatment does not prevent cell body shrinkage in the motor cortex in adult monkeys subjected to unilateral cervical cord lesion

**DOI:** 10.1186/1471-2202-9-5

**Published:** 2008-01-14

**Authors:** Marie-Laure Beaud, Eric Schmidlin, Thierry Wannier, Patrick Freund, Jocelyne Bloch, Anis Mir, Martin E Schwab, Eric M Rouiller

**Affiliations:** 1 Unit of Physiology and Program in Neurosciences, Department of Medicine, Faculty of Sciences, University of Fribourg, Chemin du Musée 5, CH-1700 Fribourg, Switzerland; 2 Brain Research Institute, Dept. Neuromorphology, University and ETH Zurich, Winterthurerstr. 190, CH-8057 Zürich, Switzerland; 3 Dept. of Neurosurgery, Neurosurgery Clinic, University Hospital of Lausanne, Rue du Bugnon, CH-1011 Lausanne, Switzerland; 4 Neuroscience Research, Novartis Institute for BioMedical Research, CH-4002 Basel, Switzerland

## Abstract

**Background:**

After unilateral cervical cord lesion at the C7/C8 border interrupting the dorsolateral funiculus in adult monkeys, neutralization of Nogo-A using a specific monoclonal antibody promoted sprouting of corticospinal (CS) axons rostral and caudal to the lesion and, in parallel, improved functional recovery. In monkeys lesioned but not treated with the anti-Nogo-A antibody, the CS neurons in the contralesional primary motor cortex (M1) survived to the axotomy, but their soma shrank. Because the anti-Nogo-A treatment induces regeneration and/or sprouting of CS axons, it may improve access to neurotrophic factors. The question therefore arises as to whether anti-Nogo-A treatment prevents the soma shrinkage observed in the contralesional M1?

**Results:**

Using the marker SMI-32, a quantitative and qualitative anatomical assessment of the pyramidal neurons in the layer V (thus including the CS cells) in M1 was performed and compared across three groups of animals: intact monkeys (n = 5); monkeys subjected to the cervical cord lesion and treated with a control antibody (n = 4); monkeys with the cervical lesion and treated with anti-Nogo-A antibody (n = 5). SMI-32 positive neurons on the side contralateral to the lesion were generally less well stained than those on the ipsilesional hemisphere, suggesting that they expressed less neurofilaments. Nevertheless, in all three groups of monkeys, the amount of SMI-32 positive neurons in both hemispheres was generally comparable, confirming the notion that most axotomized CS neurons survived. However, shrinkage of CS cell body area was observed in the contralesional hemisphere in the two groups of lesioned monkeys. The cell surface shrinkage was found to be of the same magnitude in the monkeys treated with the anti-Nogo-A antibody as in the control antibody treated monkeys.

**Conclusion:**

The anti-Nogo-A antibody treatment did not preserve the axotomized CS cells from soma shrinkage, indicating that the anti-Nogo-A antibody treatment affects morphologically the axotomized CS neurons mainly at distal levels, especially the axon collateralization in the cervical cord, and little or not at all at the level of their soma.

## Background

The motor deficits associated with interruption of the CS tract at a segmental level in monkeys were assessed in several studies [1-10]. More precisely, a surprisingly good and rapid recovery of dexterous finger movements of the ipsilateral hand took place after hemi-section at C3 level in either newborn and juvenile monkeys [4,5], or in adult monkeys after hemi-section at C4/C5 [8] or C7/C8 level [9,10]. Immediately after the cervical hemi-section and later on during the recovery, there was a dramatic reduction of the CS projection to the hemi-cord caudal to the lesion [4], indicating that the spontaneous recovery of manual dexterity was not due to a substantial reconstruction of the lesioned projection but rather to enhancement of the transmission of information from cortex to spinal cord in a reduced number of CS and/or corticobulbospinal projections together with a contribution of a more effective use of spinal circuits.

As far as the fate of the axotomized CS neurons is concerned, some controversy can be found in the literature. Some earlier anatomical studies suggested that pyramidotomy [11,12] or cervical cord lesion [6,13] induced the death of a substantial part of the large CS neurons in the contralateral primary motor cortex (M1), amounting up to 70% loss [11]. In sharp contrast, other authors concluded that there was no retrograde degeneration with breakdown and loss of neurons after section of the CS tract [14-16]. In a recent study [17], the issue of the fate of axotomized CS neurons was re-examined in two monkeys using SMI-32 as a specific marker for pyramidal neurons. We found that, after unilateral lesion of the dorsolateral funiculus at cervical level (C7-C8), the CS neurons in the contralesional primary motor cortex (M1) survived the axotomy, but their soma shrank [17].

In a recent report, evidence was provided in monkeys that the functional recovery from unilateral cervical cord lesion and CS axonal sprouting can be enhanced by an antibody treatment neutralizing the neurite growth inhibitor Nogo-A [10], extending to the primates previous results obtained in the rat [18-21]. Indeed, several functional readouts of manual dexterity showed a faster and more complete recovery of manual dexterity in a group of six anti-Nogo-A antibody treated monkeys subjected to cervical hemi-section than in a group of six monkeys subjected to a comparable lesion but treated with a control antibody [10]. Such enhancement of manual dexterity promoted by anti-Nogo-A antibody treatment was associated with an axonal sprouting of CS axons in the cervical cord rostral and caudal to the lesion [10,22]. These new fibers could provide the axotomized CS neurons an augmented access to neurotrophic factors compared to that available to the axotomized CS neurons in control antibody treated animals. The goal of the present study was thus to investigate whether the anti-Nogo-A antibody treatment also exerts an effect on the CS neurons at the level of their soma in the contralesional motor cortex, for instance by preventing the cell soma shrinkage occurring in the monkeys subjected to cervical lesion and treated with a control antibody [17]. This hypothesis is plausible because the treatment induces regeneration and/or sprouting of CS axons and may thus increase the access to neurotrophic substances. Moreover, although the anti-Nogo-A antibody was delivered intrathecally in the cervical cord close to the lesion, the antibody was shown to reach the entire brain through the CSF circulation [23] and, therefore, it may have affected the CS cell bodies in the cerebral cortex as well.

## Results

### Cervical cord lesion

**Figure 1:**
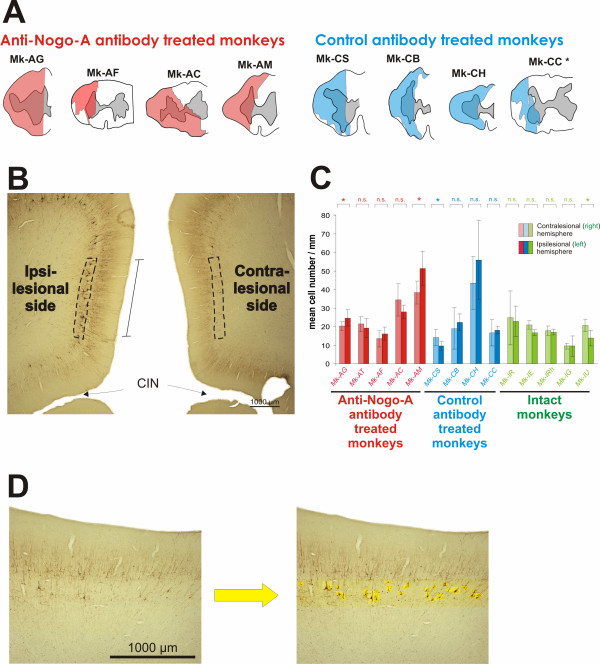
**Spinal cord lesion and number of pyramidal neurons in layer V of the motor cortex**. A: Reconstruction from paralongitudinal sections of the lesion performed in eight monkeys at cervical level C7/C8. The lesion area is indicated in red for the anti-Nogo-A antibody treated monkeys and in blue for the control antibody treated monkeys. The gray zone in the center corresponds to the gray matter. In one monkey (Mk-AT), the tissue was damaged in the zone of the cervical cord lesion and it was not possible to precisely assess the position and extent of the lesion. The lesions have already been shown in recent reports [10,22]. Although some lesions were on the left side and others on the right side, for simplification they were all represented here as a left side lesion. B: Photomicrograph showing the part of M1 corresponding to the hindlimb representation on both hemispheres and stained with SMI-32. On both sides, SMI-32 typically labeled the pyramidal cells in the layers III and V. However, typically at such low magnification, the layer V appears more densely stained on the ipsilesional hemisphere than on the contralesional one (see text). The hindlimb area in M1 was thus visible on the same section for the two hemispheres and the observer could thus delineate roughly equally long portions of the layer V in each hemisphere on which the counts were performed (dashed line), as explained in detail earlier [17]. The length of the portion of layer V in which counts were made (in mm) was determined for data normalization. CIN = cingulate sulcus. C: Bar graph indicating the mean number of SMI-32 positive neurons per unit length (and SD) counted in layer V of the hindlimb area in M1 of each hemisphere, on the sample of sections examined in the three groups of monkeys (green is for intact monkeys). * is for p <= 0.05; n.s. is for p > 0.05 (t-test for small samples as explained in the methods section). D: A portion of the hindlimb area in M1, on which morphometric measurements were conducted, is shown at higher magnification, for a typical contralesional hemisphere (left photomicrograph). At low magnification (panel B), due to lighter SMI-32 staining than in the ipsilesional hemisphere, very few SMI-32 positive neurons were visible in layer V. At this magnification (left photomicrograph), some SMI-32 positive neurons appear. The observer then identified at high magnification all SMI-32 positive neurons with a visible nucleus in layer V (neurons highlighted on top of a yellow background), as shown on the right photomicrograph. In general, it turned out that, in spite of a lighter SMI-32 staining, the positive neurons with a visible nucleus were on average as numerous as in the ipsilesional hemisphere. In each photomicrograph, the cortical surface is on top and the white matter on bottom. The SMI-32 positive neurons are typically found in layers III and V.

For the animals subjected to the unilateral spinal cord section, the extent of the lesion was assessed by reconstructing the incision site from histological sections (Fig. 1A). In most monkeys, the lesion completely transected the dorsolateral funiculus, thus corresponding to a complete transection of the CS axons originating from the contralesional hemisphere (as checked with BDA labeling of CS axons in most monkeys [10]). In one monkey (asterisk in Fig. 1A), the dorsolateral funiculus was not completely transected and therefore a few CS axons as well as RS axons were spared by the lesion. The other seven monkeys had a complete transection of the dorsolateral funiculus on the lesioned hemi-cord (Table 1), thus corresponding to a complete interruption of the main CS tract originating from the opposite hemisphere (as discussed in detail earlier in [10]). The monkeys Mk-AF and Mk-CC clearly had an incomplete lesion of the RS tract, whereas monkeys Mk-CS and Mk-CB possibly had an incomplete lesion of the RS tract (though transected at 95% at least). The RS tract transection was complete in the other four monkeys with complete lesion of the dorsolateral funiculus (Mk-AG, Mk-AC, Mk-AM and Mk-CH).

**Table 1:** List of cervical cord lesioned and intact monkeys included in the present study with identification code (same as in [10]).

	**Mk-AG**	**Mk-AT**	**Mk-AF**	**Mk-AC**	**Mk-AM**	**Mk-CS**	**Mk-CB**	**Mk-CC**	**Mk-CH**	**Mk-IR**	**Mk-IE**	**Mk-IZ**	**Mk-IRh**	**Mk-IG**
species	fasc.	fasc.	mul.	fasc.	fasc.	mul.	fasc.	fasc.	fasc.	mul.	mul.	fasc.	mul.	mul

**Antibody Treatment**	**Anti-Nogo-A **(hNogo)	**Anti-Nogo-A **(hNogo)	**Anti-Nogo-A **(11C7)	**Anti- Nogo-A **(hNogo)	**Anti-Nogo-A **(hNogo)	**Contr.**	**Contr.**	**Contr.**	**Contr.**	-	-	-	-	-
**Hemi-section total extent (%)**	**78**	*	**56**	**85**	**80**	**63**	**75**	**38**	**90**	-	-	-	-	-
**Completeness of dorsolateral funiculus section**	**Yes**	*	**Yes**	**Yes**	**Yes**	**Yes**	**Yes**	**No**	**Yes**	-	-	-	-	-
**RS and CS lesion extent (%)**	**100**	*	**73**	**100**	**100**	**87**	**93**	**61**	**100**	-	-	-	-	-
**Functional Recovery (%)**	**100**	(100)*	**57**	**100**	**96**	**22**	**78**	**83**	**53**	-	-	-	-	-
Completeness of CS/RS section	**Yes**	*	**No**	**Yes**	**Yes**	**Yes**	**Yes**	**No**	**Yes**	-	-	-	-	-
Survival time after lesion (in days)	112	97	144	135	138	198	225	105	138	-	-	-	-	-
ICMS	no	no	yes	no	no	yes	no	yes	no	no	no	no	no	no

At the time of the experiment, the lesioned monkeys had different names, not indicating whether the animal was infused with the control or the anti-Nogo-A antibody, corresponding to a double blind procedure (except Mk-AF, Mk-CS and Mk-CC). New names were assigned to the monkeys during the writing of the manuscript to improve its readability, in consistency with [10].

Under species, "mul." is for macaca mulatta while "fasc." is for macaca fascicularis.

The five anti-Nogo-A antibody treated monkeys are in the five leftmost columns ("Anti-Nogo-A") with indication of which of the two antibodies was used (mAB11C7 or mAB hNogo-A), whereas the four control antibody treated monkeys ("Contr.") are in the next four columns. The five intact monkeys are in the five rightmost columns.

Total hemi-section extent was calculated as the percentage of corresponding hemi-cord affected by the lesion on the frontal reconstruction of the cervical cord.

To tentatively determine more precisely the extent of the RS and CS lesions a quadrant of the lateral funiculus in the white matter was outlined going from the dorsal to the ventral rootlet zone. This was indicated as RS and CS lesion extent.

Functional recovery was assessed here for manual dexterity based on the "modified Brinkman board" task (see [10]), by giving in percent the ratio of the post-lesion score to the pre-lesion score.

In the row "completeness of RS section", "No" and "Yes" indicates whether the RS transection was considered as partial or as complete, respectively (for further explanation see text).

Survival time: number of days separating the cord hemi-section from the sacrifice of the animal.

* In this animal the total hemi-section extent and consequently also the RS and CS extent could not be calculated for technical reasons (poor quality of histology). In addition in this same animal the lesion was too caudal and thus the 100% functional recovery cannot be interpreted.

ICMS is for intracortical microstimulation. "Yes" means that the corresponding monkeys were subjected to extensive ICMS both pre- and post-lesion in order to study the change of motor maps (somatotopy) in relation to the lesion (see [9, 28]).

### Do some axotomized CS neurons degenerate?

In a recent paper, we reported that the number of SMI-32 positive pyramidal neurons in layer V in M1 was comparable in the contralesional and in the ipsilesional hemispheres in two monkeys subjected to an unilateral cervical lesion (treated with a control antibody), and amounted to a comparable figure as in two intact animals [17]. In the present study, these data were extended to a total of four monkeys subjected to the cervical lesion (treated with a control antibody) and five intact monkeys (Fig. 1C; blue and green bars, respectively). In the group of intact monkeys, as expected the majority (four out of five) of animals did not show a statistically significant difference of SMI-32 positive neurons between the two hemispheres (p > 0.05, n.s. in Fig. 1C). However, in one intact monkey (Mk-IU), the number of SMI-32 positive neurons per unit length was significantly higher (p < 0.05) in the right than in the left hemisphere (Fig. 1C), indicating that even in intact monkeys there may be cases in which the density of SMI-32 positive neurons in layer V of M1 differs between the two hemispheres. Accordingly, there was also one monkey (Mk-CS) in the group of lesioned monkeys treated with the control antibody (blue bars in Fig. 1C) exhibiting an inter-hemispheric difference of the number of SMI-32 positive neurons per unit length (p < 0.05), whereas in the other three monkeys the difference was not statistically significant. However, still in the group of lesioned monkeys treated with the control antibody (blue bars in Fig. 1C), there was no systematic trend with respect to the side of the lesion. These data thus confirm the notion that axotomy of the crossed CS tract at cervical level in macaque monkeys treated with a control antibody does not lead to significant neuronal loss. As anticipated, the same conclusion applies to the group of five monkeys subjected to cervical cord lesion and treated with anti-Nogo-A antibody (Fig. 1C; red bars): in two monkeys there were slightly more SMI-32 positive neurons in the contralesional hemisphere whereas in the other three monkeys the ipsilesional hemisphere had more SMI-32 positive neurons (in two of them, the difference was statistically significant, but to a similar extent as in the case observed in each of the other two groups of monkeys). Overall, the unsystematic inter-hemispheric differences in the two groups of lesioned monkeys were generally in the same order of magnitude as those observed in the group of intact monkeys.

### Does anti-Nogo-A treatment prevent shrinkage of CS neurons after axotomy?

**Figure 2:**
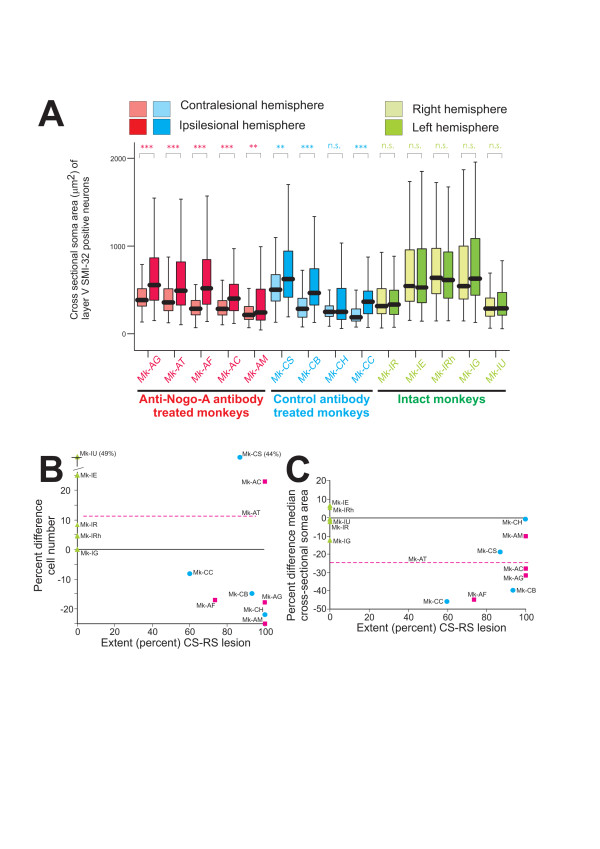
**Somatic size of pyramidal neurons in layer V of the motor cortex and inter-hemispheric differences**. A: Box and whisker plots showing the distribution of somatic cross-sectional areas of SMI-32 positive neurons in layer V in M1 for the three groups of monkeys. In the box and whisker plots, the thick horizontal line in the box corresponds to the median value, whereas the top and bottom of the box are for the 75 and 25 percentile values respectively. The top and bottom extremities of the vertical lines on each side of the box are for the 90 and 10 percentile values, respectively. All but one lesioned monkey (red and blue bars) exhibited a significant inter-hemispheric difference of cross-sectional soma area (* = p < 0.05; ** = p < 0.001; *** = p < 0.0001; Mann and Whitney test), whereas in the intact monkeys the difference was not statistically significant (n.s. = p > 0.05; Mann and Whitney test). B: Inter-hemispheric percent difference in the number of SMI-32 positive neurons per unit length in layer V in M1 for the same three groups of monkeys as in Figure 1C (same color code), plotted as a function of the lesion extent (percent of the territory corresponding to the CS and RS tracts affected by the lesion). Note that the differences are not systematic with respect to the side of the lesion in the two groups of lesioned animals (red squares and blue circles for the anti-Nogo-A and control antibody treated monkeys, respectively). In the percent comparison, 100% is for the number of SMI-32 positive neurons per unit length on the ipsilesional side (on the left side for the intact monkeys). The green triangles are for the intact monkeys. As the lesion extent could not be determined for Mk-AT (see Table 1), its corresponding percent value was represented by an horizontal dashed line. C: Inter-hemispheric percent difference of the median value of the cross-sectional soma area of SMI-32 positive neurons in layer V in M1 for the same three groups of monkeys as in panel A, plotted as a function of the lesion extent (percent of the territory corresponding to the CS and RS tracts affected by the lesion). Note that the difference is small for the intact monkeys (green symbols), but it is more prominent and systematic with respect to the side of the lesion for most lesioned monkeys, with considerable overlap between the two groups of lesioned monkeys. Same conventions as in panel B.

In our recent study [17], the two monkeys subjected to the cervical lesion and treated with the control antibody exhibited a shrinkage of their soma as a result of the CS axotomy at cervical level. This observation is confirmed here on a larger pool of four control-antibody treated monkeys (Fig. 2A; blue bars): in three of the four monkeys, the SMI-32 positive neurons of layer V were significantly smaller in the contralesional hemisphere than in the ipsilesional one. In the fourth monkey (Mk-CH), no somatic size difference was found, but in this particular animal the quality of the SMI-32 immunostaining was poor, which may in part explain this result. In contrast to the majority of control antibody treated monkeys, the intact monkeys did not show a statistically significant difference in the distribution of somatic area of layer V pyramidal neurons between the two hemispheres (Fig. 2A; green bars). It can be concluded that CS axotomy induced shrinkage of somatic area of the corresponding CS neurons in the lesioned monkeys treated with the control antibody.

To address the issue whether anti-Nogo-A antibody treatment, in addition to enhanced sprouting in the cervical cord near the lesion [10,22], also prevents shrinkage of the soma of the CS axotomized neurons, the same analysis was conducted here in the group of five anti-Nogo-A antibody treated monkeys. In all five monkeys (Fig. 2A; red bars), the soma cross-sectional areas of SMI-32 positive layer V pyramidal neurons were significantly reduced in the contralesional hemisphere as compared to the ipsilesional one, indicating that the CS axotomized neurons shrank. Moreover, the extent of shrinkage is generally comparable in the anti-Nogo-A antibody treated and control antibody treated monkeys (Fig. 2C). In other words, the anti-Nogo-A antibody treatment did not prevent or reduce shrinkage of the soma of axotomized CS neurons.

The data of the present study are summarized in Figures 2B and 2C, where the inter-hemispheric difference (in percent) of the number of SMI-32 positive neurons per unit length in the layer V of M1 and of the median of their cross-sectional soma area were plotted as a function of the extent of the lesion, estimated in percent of the lateral spinal cord. For the three groups of monkeys, there was no systematic inter-hemispheric difference for the number of SMI-32 positive neurons per unit length in layer V of M1 (Fig. 2B), indicating that the unilateral axotomy of the CS tract did not produce a substantial cell loss in the contralesional hemisphere. Indeed, in the two groups of lesioned monkeys, the inter-hemispheric cell number difference was in the same range as in intact monkeys. On the other hand, as compared to intact monkeys, the cervical hemi-section led to a significant shrinkage of SMI-32 positive cells in layer V of the contralesional M1, irrespective of whether the monkey was treated with a control antibody or an anti-Nogo-A antibody (Fig. 2C).

## Discussion

Based on a substantially increased number of animals, the present study confirms preliminary data [17] indicating that unilateral cervical lesion is not followed by a significant elimination of axotomized CS neurons. This conclusion thus adds a new piece of evidence to the long debated issue of whether CS neurons die after axotomy in monkeys. The present data are in line with previous reports claiming that there is no extensive CS cell loss after spinal cord lesion in monkeys [14-16]. As compared to the studies which previously addressed this question, the present investigation is based on a specific staining of pyramidal neurons in layer V (SMI-32), allowing better focus on the neurons of interest (CS neurons) than for instance in Nissl staining, as used in most cases previously. Although SMI-32 stains pyramidal cells in layer V in general and not only the CS neurons, the measurements conducted in the present study are thus somewhat more directed towards the subpopulation of CS neurons than measurements conducted for instance in Nissl material, where a much larger population of neurons would be involved. As a consequence, a difference between the two hemispheres affecting the CS neurons after a unilateral cervical lesion is detected with more sensitivity in SMI-32 than in Nissl material. Our measurements thus include the large pyramidal neurons of layer V in the motor cortex, which in the rat primary somatosensory (barrel) cortex express intraneuronal Nogo-A [24]. Whether this is also true in primates and relevant in the present context remains an open question.

To investigate whether cell death occurred in layer V in the contralesional hemisphere, we counted the number of SMI-32 positive neurons in layer V in M1 territories of roughly comparable size delineated on the same section in the two hemispheres. Moreover, the data were normalized with respect to the length of the layer V territory in which the measurements were conducted. Our goal here was not to establish the absolute number of SMI-32 positive neurons in a given cortical area but rather to compare between the two hemispheres their relative numbers. Furthermore, because the number of SMI-32 stained neurons in the delineated territory of interest was relatively low, we did not use a stereological probe to estimate their number, rather we counted all of them exhibiting their nucleus. This approach is considered as adequate for studies dealing with few neurons [25-27]. For example, in a recent morphometrical study in the hippocampus [25], the authors used a true stereological approach to estimate the volume of the structure of interest as well as the total absolute number of neurons. In contrast, to estimate the number of BrdU immunoreactive neurons, the authors did not use a stereological probe because "of the low number of BrdU-labelled cells [25]." In the present case, the number of SMI-32 positive neurons with a visible nucleus in the restricted zone of interest of M1 (layer V) was also very low, thus preventing the use of stereological probe. Furthermore, the data and their reproducibility obtained in the five intact monkeys strongly support the validity of the method used here to establish the number of SMI-32 positive neurons and their cross-sectional somatic area. Indeed, in all five intact monkeys, there was no statistically significant inter-hemispheric difference for their soma area. Furthermore, the number of SMI-32 positive cells did not show substantial inter-hemispheric difference in four out of five intact monkeys. In contrast, the somatic area difference observed in the two groups of lesioned monkeys was considerable and systematic with respect to the side of the lesion (except in one animal, Mk-CH). The morphometrical measurements were conducted in the hindlimb area of M1 and not in the hand area because the latter zone was subjected, in some animals, to extensive intracortical microstimulation [9,28] (see also Table 1) and, in all animals, to the injection of an anterograde tracer to label the CS tract [10,22]. The hand area in M1 was thus not suitable for an optimal detection of SMI-32 positive neurons as they may be obscured by the presence of the reaction product corresponding to the injection site. The hindlimb area, also affected by the cervical lesion, was thus more appropriate to conduct the anatomical analysis and we believe that the present data derived from the CS neurons of the hindlimb area is very likely to apply also to the CS neurons of the hand representation.

As shown in Figure 1C, there was no systematic inter-hemispheric difference in the number of SMI-32 positive neurons in the three groups of monkeys, thus supporting the conclusion that the cervical cord hemi-section did not provoke substantial loss of axotomized CS neurons. Considering the results in Figure 1C for the two groups of monkeys subjected to the cervical cord hemi-section (red and blue bar graphs), surprisingly three monkeys (Mk-AT, Mk-AC and Mk-CS) exhibited a higher number of SMI-32 labeled neurons on the contralesional hemisphere. In the first two monkeys, this difference was however not statistically significant, reflecting in part the variability of the method. For the third monkey (Mk-CS), the significant difference may reflect an episodic interhemispheric difference in a minority of monkeys, as actually observed in one intact monkey (Mk-IU in Fig. 1C) out of five intact animals. Nevertheless, the presence of some lesioned monkeys with more SMI-32 positive neurons on the contralesional hemisphere reinforces the conclusion of an absence of elimination of CS axotomized neurons after cervical cord hemi-section. Overall, as shown in Figure 1C, the number of SMI-32 positive neurons varied across animals, most likely reflecting individual variability both in the number of large layer V pyramidal neurons and in the intensity of SMI-32 staining.

The observation that there was no substantial loss of axotomized CS neuron after cervical hemi-section represents a quite favorable outcome for functional recovery. Indeed, the axons that have been severed, although they retract some distance from the lesion [22], are still present and potentially in position to regenerate or give rise to collateral sprouting. In other words, the functional recovery does not depend only on CS neurons spared by the lesion or on other preserved descending tracts. In recent reports, we demonstrated that the severed CS axons indeed can give rise to spontaneous axonal sprouting in absence of treatment, but only to a very limited extent [10,22]. In contrast, an anti-Nogo-A antibody treatment substantially enhanced CS axonal sprouting from the severed axons, as manifested by an increase of axonal arbors rostral to the lesion as well as axonal arbors and swellings caudal to the lesion [10,22].

As a result of cervical cord hemi-section, the axotomized CS neurons in the contralesional hemisphere exhibit shrinkage of their soma (Fig. 2A, blue bar graphs). Neuron atrophy in response to axotomy is very well known and widely observed, including cortical neurons. Neuronal metabolism depends on continuous retrograde trophic signals from the targets, which are absent or reduced in case of axotomy. As the normal intact axonal arborization may be much larger than the "spontaneously" newly formed arbor of the few sprouting neurons in the control antibody treated monkeys, the decrease of retrogradely transported trophic signals would thus contribute to the soma shrinkage. In the anti-Nogo-A antibody treated monkeys, although sprouting was enhanced [22], the quality and amount of trophic signals may be still insufficient to completely reverse the atrophy. Furthermore, the number of CS axons that successfully sprout and re-establish an axonal arbor with contacts is not precisely known. It is likely that only a relatively limited proportion of axotomized CS neurons managed to re-establish a large axonal arbor and to obtain enough trophic signals to prevent or reverse the atrophy. Increase of the somatic size of only of few neurons would remain undetected in the soma size distribution (Fig. 2A). Finally, the time course has to be taken into consideration. Shortly after the lesion, sprouting neurons are not in an atrophic state and this is true for the initial few weeks after the injury when sprouting takes place. The present morphometric measurements were conducted in our monkeys many months post-lesion at a time point when sprouting was completed. However, as the newly formed axonal arbors may not be as large as intact ones, the total amount of trophic support they receive may still not be enough to maintain their full soma size.

The present study provides evidence that the anti-Nogo-A antibody treatment limits its action on the distal part of the axotomized CS neurons, namely by enhancing axonal sprouting close to the lesion itself, but does not prevent changes taking place at the level of the CS cell body, at least as far as soma size shrinkage is concerned. This conclusion applies to the present experimental conditions and it cannot be excluded that soma shrinkage of axotomized CS neurons could have been better prevented, at least to some extent, if the dose of the anti-Nogo-A antibody would have been higher or if it would have been delivered not only near the cervical lesion but also at cortical level. Indeed, although the anti-Nogo-A antibody delivered intrathecally near the lesion site penetrates into the CNS, the penetration was less complete in the brain than in the spinal cord [23].

To our knowledge, the issue of CS neurons elimination after spinal cord lesion and treatment with anti-Nogo-A antibody has not been investigated before, even in rodents and therefore the present observation of an absence of anti-Nogo-A antibody treatment effect on the shrinkage of axotomized CS neurons is original. The issue of whether the anti-Nogo-A antibody treatment can contribute to rescue cells from elimination after axotomy could not be addressed here as there was no CS neuron loss, even in the control antibody treated monkeys. The absence of CS cell loss after cervical lesion contrasts with the number of RS neurons detected in the contralesional magnocellular red nucleus (RNm), which was up to 30% lower than in the ipsilesional RNm [29]. However, in the latter study conducted on the same groups of lesioned monkeys, it was observed that the anti-Nogo-A antibody treatment did not impact on the RS neurons, as the number of detected RS neurons was comparable in the anti-Nogo-A antibody and in the control antibody treated monkeys.

## Conclusion

In adult macaque monkeys, following cervical cord hemi-section, neutralization of Nogo-A with a specific monoclonal antibody enhanced functional recovery [10] and promoted sprouting of the corticospinal tract both rostral [22] and caudal to the lesion [10,22], as compared to monkeys treated with a control antibody. In the control antibody treated monkeys, the corticospinal neurons in the contralesional primary motor cortex (M1) survived to the axotomy, but their soma shrank. The present study tested the hypothesis that the anti-Nogo-A treatment may prevent such soma shrinkage. We found that the anti-Nogo-A antibody treatment did not preserve the axotomized corticospinal cells from soma shrinkage, indicating that the anti-Nogo-A antibody treatment affects morphologically the axotomized corticospinal neurons mainly at distal levels, especially the axon collateralization in the cervical cord, and little or not at all at the level of their soma.

## Methods

The present data have been derived from a long-term protocol, described earlier in detail [9,10,17,22,28], conducted on monkeys subjected to unilateral cervical cord lesion at the C7/C8 border, in accordance with the Guide for the Care and Use of Laboratory Animals (ISBN 0-309-05377-3; 1996) and approved by local (Swiss) veterinary authorities. The present study aimed at comparing three groups of monkeys (Table 1): (i) Intact monkeys (n = 5); (ii) Monkeys subjected to the cervical hemi-section and treated with anti-Nogo-A antibody (n = 5); (iii) Monkeys subjected to the cervical hemi-section and treated with a control antibody (n = 4).

Two monoclonal antibodies (mAbs) against different sites of the neurite inhibitor protein Nogo-A were employed (Table 1; see also [22] for more detail on the antibodies) in the group of anti-Nogo-A antibody treated monkeys: the mouse mAB 11C7 was raised against a 18 amino acid sequence of rat Nogo-A (aa623 – 640) close to the most inhibitory region of the Nogo-A protein [30]. The second antibody used, mAb hNogo-A recognized the Nogo-A specific region of the human Nogo-A sequence. Both antibodies identify primate Nogo-A monospecifically on Western blots [23,30]. The antibodies were purified as IgGs and concentrated to 3.7–10 mg/ml in PBS. In the control antibody treated monkeys, purified IgG of a mouse mAb directed against wheat auxin (AMS Biotechnology, Oxon/UK) was used as control antibody (concentration: 3.7–10 mg/ml).

Briefly, the main steps of the protocol conducted on the lesioned monkeys were the following. First, an intensive pre-lesion training was initially performed in order to establish a stable behavioral score in manual dexterity tasks involving both hands [10]. A unilateral lesion was performed at the C7/C8 border [10,17] and the antibody (either control or anti-Nogo-A) was administered intrathecally a couple of mm rostral to the lesion. The manual dexterity tests were conducted at regular intervals for several months post-lesion, until the animals reached a plateau reflecting a stable level of functional recovery. Anterograde tracers were injected mainly in the hand representation of the primary motor cortex (M1) bilaterally to label the CS tract [10,22]. The monkeys were sacrificed under deep anesthesia and perfused transcardially with fixatives [17]. Frozen sections comprising each of the two hemispheres were cut in the coronal plane at a thickness of 50 μm. The spinal cord was cut in the paralongitudinal plane at the site of the lesion and transversally at the level of the first cervical segments as well as of thoracic segments caudal to the lesion. The lesion was reconstructed using SMI-32 stained material in all monkeys as previously reported [10,17] and its extent was expressed quantitatively by the percent of the territory delimited by two lines starting from the central channel and extending to the dorsal and ventral rootlets, thus covering completely the lateral funiculus and hence the zone occupied by the decussated CS and rubrospinal (RS) tracts. A series of brain and spinal cord sections was treated immunocytochemically with SMI-32 antibody in order to visualize in the cerebral cortex the layers III and V pyramidal neurons (Fig. 1B), as recently described in detail [31]. The epitope recognised by the SMI-32 antibody lies on non-phosphorylated regions of neurofilament protein and is only expressed by specific categories of neurons [32,33]. SMI-32 positive neurons (only those exhibiting the nucleus) in the primary motor cortex (M1) on both hemispheres were counted under the microscope at high magnification (400×), in a territory including layer V at the same dorso-ventral location and defined of roughly equal length on both hemispheres (zone delineated with a dashed line in Fig. 1B). The typical appearance of SMI-32 positive neurons in layer V of M1 was illustrated in a recent report (see Figure 3 in [17]). At low magnification, as staining was stronger on the ipsilesional hemisphere, the number of SMI-32 positive neurons seems to be lower on the contralesional side (Fig. 1B). This is however a wrong impression as, at higher magnification (Fig. 1D), several SMI-32 positive neurons appear on the contralesional hemisphere (Fig. 1D): at high magnification, even lightly stained SMI-32 positive neurons can reliably be detected. On each section and separately for each hemisphere, the total number of SMI-32 positive neurons was divided by the length (in mm) of the territory in which the analysis was conducted (line segment in Fig. 1B). Moreover, the somatic cross-sectional silhouette area of these SMI-32 positive neurons in layer V was determined, as described in detail earlier [17]. In each monkey, four to six coronal histological sections were taken along the rostrocaudal extent of M1 (separated by 800 μm each) in which the analysis of SMI-32 positive neurons in layer V was conducted.

The number of SMI-32 positive neurons per unit length (mm) obtained in a given section was averaged across the sections analyzed in each monkey, separately for the two hemispheres, and a standard deviation was obtained reflecting the variability from one section to the next (Fig. 1C). This measure is proportional to the number of SMI-32 positive neurons per volume unit. The statistical comparison of the number of SMI-32 positive neurons per unit length between the two hemispheres was based on a paired t-test for small samples applied on the four to six sections analyzed in each monkey. A statistically significant difference of the number of SMI-32 positive neurons in layer V between the two hemispheres was obtained for a t value corresponding to p <= 0.05 (df = 3 to 5 for 4 to 6 sections analyzed, respectively). The soma areas did not follow a normal distribution (wider dispersion for large somatic areas than small ones) and therefore they were graphically represented in the form of box and whisker plots (putting emphasis on the median value rather than the mean value; Fig. 2A). Accordingly, the statistical comparison of soma areas of SMI-32 positive neurons between the two hemispheres was conducted for each animal using the non-parametric unpaired Mann and Whitney test (Fig. 2A), with a significance level of p <= 0.05.

## List of abbreviations

CS = corticospinal

M1 = primary motor cortex

Mk = monkey

RNm = magnocellular red nucleus

RS = rubrospinal

SCI = spinal cord injury

## Authors' contributions

EMR designed the study, contributed to the experiments and analysis of the data, and drafted the manuscript. MLB conducted the morphological measurements in most monkeys, contributed to the analysis and wrote a preliminary version of the manuscript in the context of her Master thesis. ES and TW designed the study, carried out the experiments, the morphological measurements on some monkeys and analyzed the corresponding data. PF contributed to the experiments and the analysis of the data. JB designed the study and performed the cervical lesions. AM contributed to the study design and antibody protocol. MES contributed to the study design and general concept, antibody protocol and data analysis. All authors read, commented and approved the final manuscript.
